# Risk Assessment and Optimization for Pregnancy in Patients with Rheumatic Diseases

**DOI:** 10.3390/diagnostics14131414

**Published:** 2024-07-02

**Authors:** Alyssa Kwok

**Affiliations:** Department of Internal Medicine, Dell Medical School, The University of Texas at Austin, 1601 Trinity St., Austin, TX 78712, USA; alyssa.kwok@austin.utexas.edu

**Keywords:** adverse pregnancy outcomes, fertility, medication safety, risk factors, rheumatic disease, disease-modifying antirheumatic drug

## Abstract

Patients with rheumatic diseases frequently operate with incomplete or incorrect information while planning for and experiencing pregnancy, often due to variability in provider care and knowledge. Risk assessment at each stage of pregnancy—pre-conception, during pregnancy, and postpartum—is focused on reducing maternal and neonatal complications. This review aims to compile updated, evidence-based guidance on how to minimize risk factors contributing to adverse pregnancy outcomes (APOs). Mitigation of known causes of infertility, appropriate testing and monitoring, achieving low disease activity on pregnancy-safe disease-modifying antirheumatic drugs (DMARDs) prior to conception, controlling hypertension (a frequent comorbidity among patients with certain rheumatic diseases), and the use of appropriate adjunctive medications (such as low-dose aspirin when preeclampsia risk is high) can optimize fertility and prevent adverse maternal and neonatal outcomes.

## 1. Introduction

Whether or not a patient desires pregnancy in the near future is a foundational question for rheumatologists. It affects testing, fertility counseling (and the possible need for preservation), choice of medications, and monitoring for complications. A patient’s answer to this question may change throughout their journey with the clinician; therefore, revisiting this question frequently is important in the care of patients with childbearing ability. 

A great unmet need exists in this area. Patients would like to have frequent discussions, initiated well before conception, about how to plan for and manage their disease during pregnancy [1]. In the absence of such information, patients have disclosed trying to fill the gap with social media and online forums [2]. Two mixed-methods studies [2,3] from England and Australia demonstrated the difficulty that women with rheumatic disease encounter when trying to obtain the information they need in order to make informed decisions surrounding family planning, pregnancy, and the postpartum period. From their accounts in qualitative interviews, more help is needed for the timing of conception and medication changes. Concerns included how medication changes might affect disease activity, pregnancy, and breastfeeding. Other issues included lack of access to evidence-based pre-conception counseling and multidisciplinary care. There were fears about the negative impact of increased disease activity on the ability to care for one’s child, with a subsequent increased need for childcare and social and psychological support. Iranian patients shared these fears, along with experiences of healthcare professionals who advised against pregnancy, did not communicate well with respect to reproductive decisions, and were unwilling or unable to provide answers to patients’ questions in this area [4]. Patients surveyed in Japan reported avoiding pregnancy due to concerns about the risk of their medications on the fetus and a fear of hereditary transfer of their disease to offspring [5]. Armed with either information, a lack thereof, or misinformation, patients are struggling to make life-altering reproductive decisions in this challenging milieu. 

Unfortunately, rheumatology providers are not always up to date on how to properly screen, risk-stratify, and optimize care for patients desiring pregnancy. A study of 270 rheumatologists who were anonymously surveyed demonstrated a gap in knowledge surrounding teratogenic medications and the efficacy of various contraceptive methods [6]. This group included providers at rheumatology meetings and major academic centers. Among other deficiencies, only 37% were able to identify mycophenolate as a teratogen, 69% cyclophosphamide, and 88% methotrexate. Birru Talabi et al. found that about 80% of patients reported discontinuing medications that would have been safe in pregnancy and lactation, and 15% of pregnancies were exposed to methotrexate, a teratogen [7]. Taken together, the risks of this confusion are real.

Even when providers have some level of awareness on this topic, patients frequently pose valid questions about the risks to their bodies and their offspring, and the answers may not always be well known, even if present in the literature. The goal of this article is to address these common issues and to assist the busy clinician in the most user-friendly way possible. As such, the article is presented in a question-and-answer format and organized based on the patient’s timeline, with a focus on the most recent literature. 

## 2. Screening

### 2.1. How Often Should We Discuss Reproductive Health? How Do We Begin?

Tools such as the One Key Question^®^ (OKQ) from Oregon and the Family Planning Quotient (FPQ) from Cook County have been developed to help facilitate contraceptive counseling and pregnancy planning [8]. Implementation of the OKQ (“Would you like to become pregnant in the next year?”) has been shown to increase rates of contraceptive counseling in primary care [9] and has been piloted in rheumatology clinic environments [10]. 

Even without a formal trademarked tool, implementing some form of standardized, regular screening to ask about a patient’s reproductive goals can be helpful in directing the conversation. The quality improvement project by Mruk and colleagues had significant decreases in unintended pregnancies by combining medication consents annually with questions about reproductive health at each visit [11].

### 2.2. How Do I Prevent Pregnancy If I’m Not Ready to Become Pregnant Anytime Soon or If I’m on a Teratogenic Medication? 

Clear guidance exists on the types of contraceptives recommended, depending on the disease and its comorbidities [1]. With a history of systemic lupus erythematosus (SLE), antiphospholipid antibody positivity, or history of blood clot, contraceptives containing estrogen should not be used. In those with osteoporosis, Depo-Medrol should be avoided. Otherwise, using the most effective contraceptive available is what matters most to prevent an unintended pregnancy. Generally, long-acting reversible contraception (LARC), such as subdermal progestin implants (e.g., Nexplanon) or intrauterine devices (IUDs, e.g., progestin or copper), are recommended for all because they can be in place for 3–10 years (depending on the type of LARC), have a 1-year failure rate of less than 1%, and do not contain estrogen. Progestin-only pills are also recommended for all patients with rheumatic disease, but their efficacy is lower, with a 1-year failure rate of 5–8%, due to the need to take the pill daily at the same time for maximum effect. In 2023, the FDA approved the first over-the-counter oral contraceptive, the Opill^®^, with the hopes of improving access to contraceptives and preventing unintended pregnancies [12]. This contraceptive, containing only progestin, is also safe to take for all rheumatic patients. 

For those on teratogenic medications, such as methotrexate, mycophenolate, cyclophosphamide, or warfarin, it is especially important to discuss contraceptive plans. Of note, if the patient takes mycophenolate (MMF) and an IUD is not in place, two forms of contraception are necessary due to hormonal changes caused by MMF [1].

A helpful summary chart of the various contraceptive methods with their safety and efficacy is included in the 2020 American College of Rheumatology reproductive guidelines.

Preventing unintended pregnancies can be challenging despite the well-documented risks. Mruk et al. reported remarkable success in avoiding pregnancy among teens taking teratogenic medications [11]. This multiyear quality improvement project utilized a combination of annual medication consents in the first phase followed by assessing sexual history in the second phase. Questions were posed at each visit regarding prior sexual activity, their desire to become pregnant within the year, and plan for birth control. Although this took place in the pediatric population, this is a model for any rheumatology practice. 

## 3. Fertility

### 3.1. Is Female Fertility Affected by Rheumatic Disease? 

Individuals with rheumatic disease can experience menstrual irregularities ranging from oligomenorrhea to menorrhagia [13]. The causes can be multifactorial and include disease activity and medications. Active inflammation, significant steroid use (which can disrupt the hypothalamic–pituitary–gonadal axis), and concomitant diseases, such as hypothyroidism or endometriosis, can also result in anovulation and amenorrhea [13,14,15]. Having irregular or absent periods can make planning a pregnancy very difficult.

Anti-Mullerian hormone (AMH) levels can reflect ovarian reserve. Patients with rheumatoid arthritis (RA), Behçet’s disease, and spondyloarthritis (SpA) have been found to have lower AMH levels compared to age-matched controls [16]. Major organ involvement (mainly renal or neurological) in SLE has been linked with lower AMH levels but having only joint or skin involvement did not seem to be associated [17].

Fertility rates have been reported to be lower with certain rheumatic diseases. For example, patients with RA, axial spondyloarthritis (axSpA), juvenile idiopathic arthritis (JIA), and other autoimmune arthritides in Scandinavian countries were more likely to be nulliparous with increased time to pregnancy over healthy controls and to have lower fertility rates after rheumatic diagnosis [18,19], but it is unclear why. Higher maternal age, active inflammation, incompatible or fertility-altering medications such as non-steroidal anti-inflammatory drugs (NSAIDs), altered sexual function, and personal preference in the context of disease-related concerns have all been hypothesized [20,21,22]. In a Norwegian study of axSpA and RA, the median time to pregnancy was about 2–3 months, with 21–24% taking over 12 months to become pregnant. Factors associated with longer time to pregnancy included age, duration of disease, and being nulliparous [19]. Patients with Sjögren’s syndrome and Behçet’s disease have not been found to have fewer pregnancies compared to controls [15,23]. Poorly controlled, longstanding familial Mediterranean fever (FMF) can cause infertility, but with well-controlled disease and treatment with colchicine, it has not been found to decrease fertility [24,25]. 

Patients with SLE can have a wide array of autoantibodies, including antiphospholipid antibodies (aPLs), which can affect ability to carry a pregnancy to term, as well as antibodies directed against ovaries, which can cause an autoimmune oophoritis [14]. Treatment with cyclophosphamide for organ-threatening disease can result in premature ovarian failure, resulting in infertility. In a 2022 systematic review, other factors found to be associated with lower fertility rates included being older at the start of SLE therapy, a lower glomerular filtration rate at the start of therapy, and a genotype (CYP2C19*1) linked to higher rates of premature ovarian failure after cyclophosphamide [26]. Although fertility can be decreased in various rheumatic diseases, this may not be the case in well-controlled SLE without the above risk factors. In a national registry of patients from Norway, SLE patients from 2006–2016 were found to have higher rates of fertility compared to patients with RA (88.7% vs. 72.2%), a difference that persisted even after adjusting for age [27]. 

Crystalline arthropathies, such as gout and calcium pyrophosphate deposition (CPPD) disease, are not as common in the premenopausal population; therefore, there are no studies in the literature on fertility rates among patients with these diseases [28,29].

### 3.2. Is Male Fertility Affected by Rheumatic Disease? 

A systematic review published in 2016 reflected the known fertility issues with cyclophosphamide but also showed possible impairment in SLE, RA, and dermatomyositis [30]. The included studies (with 5–35 patients per study) showed gonadal, sperm, and hormonal changes in patients with SLE and dermatomyositis. Anti-sperm antibodies in SLE and RA were reported in several of the studies from this review; however, the clinical significance and actionability of these antibodies are not yet clear [31]. Interestingly, aneuploidies such as Klinefelter syndrome (47,XXY) have been found to be more common among male SLE patients [32], and this is a known cause of infertility, with limited options to preserve fertility that require specialized care, counseling, and timing [33,34]. While one systematic review asserted that rheumatic disease did not appear to worsen fertility compared to controls in ankylosing spondylitis (AS) and Behçet’s disease (BD) [30], another suggested that poor semen quality and higher frequency of varicocele may pose infertility in AS and BD patients [35]. Neither gout nor treatment with colchicine seem to affect male fertility [30].

Even if comparable to the general population, infertility is due to male causes in up to 30%. This can be due to a variety of factors not related to rheumatic disease and is often idiopathic. Formal evaluation is indicated after regular unprotected sex with a partner of childbearing ability (or after 6 months of trying with a partner greater than 35 years of age) but can be pursued earlier if desired [36]. 

## 4. Planning for Pregnancy

**Figure 1:**
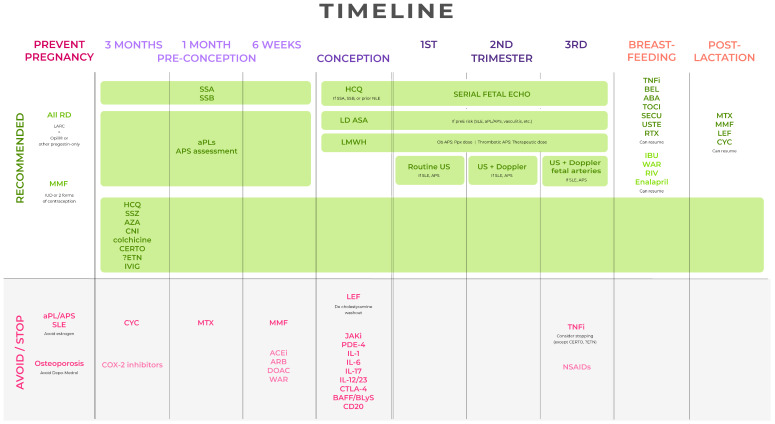
Proposed algorithm and timeline for the care of birthing patients with rheumatic disease. RD = Rheumatic disease; LARC = long-acting reversible contraceptive; MMF = mycophenolate mofetil, mycophenolic acid; IUD = intrauterine device; aPL = antiphospholipid antibody; APS = antiphospholipid syndrome; SLE = systemic lupus erythematosus; CYC = cyclophosphamide; MTX = methotrexate; LEF = leflunomide; JAKi = JAK inhibitors (e.g., tofacitinib, ruxolitinib, baricitinib); PDE-4 = PDE-4 inhibitors (e.g., apremilast); IL-1 = IL-1 inhibitors (e.g., anakinra, canakinumab, rilonacept); IL-6 = IL-6 inhibitors (e.g., tocilizumab, sarilumab); IL-17 = IL-17 inhibitors (e.g., secukinumab, ixekizumab); IL-12/23 = IL-12/-23 inhibitors (e.g., ustekinumab); CTLA-4 = CTLA-4 inhibitors (e.g., abatacept); BAFF/BLyS = BAFF/BLyS inhibitors (belimumab); CD20 = CD20 inhibitors (e.g., rituximab), TNFi = TNF inhibitors; CERTO = certolizumab; ETN = etanercept; BEL = belimumab; ABA = abatacept; TOCI = tocilizumab; SECU = secukinumab; USTE = ustekinumab; RTX = rituximab; HCQ = hydroxychloroquine; SSZ = sulfasalazine; AZA = azathioprine (up to 2 mg/kg/day); CNI = calcineurin inhibitor (e.g., cyclosporine, tacrolimus); IVIG = intravenous immunoglobulin; ACEi = angiotensin-converting enzyme inhibitors; ARB = angiotensin receptor blockers; WAR = warfarin; LMWH = low-molecular-weight heparin; DOAC = direct oral anticoagulants; NSAIDs = nonsteroidal anti-inflammatory drugs; IBU = ibuprofen; RIV = rivaroxaban; SSA = anti-Ro antibody; SSB = anti-La antibody; LD ASA = low-dose aspirin; preE = preeclampsia; Ob APS = obstetric antiphospholipid syndrome; Thrombotic APS = thrombotic antiphospholipid syndrome; NLE = neonatal lupus.

A proposed care pathway synthesizing the available guidelines and recommendations from this review is included (Figure 1). It contains guidance on testing, monitoring, and a timeline for medication changes, as well as contraception options if pregnancy avoidance is the goal.

### 4.1. What Can I Do to Reduce the Risk of Bad Outcomes? 

A major protective factor for all patients with rheumatic disease is well-controlled disease. 

A 2021 retrospective multicenter study of 347 pregnancies in 281 SLE patients demonstrated fewer flares and adverse pregnancy outcomes among those who were in remission at the beginning of pregnancy [37]. Adherence to hydroxychloroquine has been shown to be associated with fewer flares and adverse pregnancy outcomes [38]. 

In addition to stopping teratogens or medications with limited data in pregnancy, discontinuing or tapering steroid dose to the lowest effective dose, ideally under 10–20 mg daily, can also help to reduce the risk of pregnancy hypertension, gestational diabetes, and adverse pregnancy outcomes [1,38,39,40]. Low-dose aspirin should be initiated in those with a high risk for preeclampsia, and heparin or LMWH in those with APS [1,38,41]. 

Hypertension—in the context of systemic vasculitis, SLE, or on its own—increases the risk of adverse pregnancy complications [38,42]. Therefore, any comprehensive care plan for those at risk should include hypertensive control with pregnancy-safe medications.

### 4.2. Are There Special Lab Tests I Should Get? 

All rheumatic patients planning to become pregnant should have aPLs checked early (as this affects contraceptive choice) and SSA/Ro, and SSB/La antibodies should be tested prior to conception [1]. 

SSA/Ro antibodies, in particular, have been associated with risk for neonatal lupus (NLE), which can cause congenital heart block [1,38]. To decrease the risk in pregnant individuals who are SSA-positive or those with a history of NLE, hydroxychloroquine should be initiated during pregnancy and fetal echocardiograms should be obtained up to once a week starting at week 16 through to week 26. If fetal heart block (1st or 2nd degree) is present, dexamethasone can be used to prevent progression to complete heart block.

aPLs have been linked to an increased risk of adverse pregnancy outcomes, including miscarriages, fetal loss, preeclampsia, and placental insufficiency. Lupus anticoagulant, in particular, may be correlated more closely with fetal and neonatal death and preterm delivery due to placental etiologies [43]. Positivity of aPLs should prompt the consideration of low-dose aspirin, which is discussed below. 

While certain tests, such as a decreasing/low placental growth factor (PIGF) or increasing/high soluble fms-tyrosine kinase molecule-1 (sFlt-1) and soluble endoglin (sEng), can serve as biomarkers of preeclampsia, these are not specific to rheumatic disease [44]. In patients with SLE or antiphospholipid antibody positivity, these two biomarkers were linked to severe adverse pregnancy outcomes [45]. Although commercially available, they have not been widely adopted at this time [44]. 

### 4.3. What Other Monitoring Do I Need? 

Due to adverse pregnancy outcomes associated with active SLE, the ACR has recommended monitoring for disease activity at least once a trimester [1]. EULAR has also recommended fetal ultrasounds during each trimester for those with SLE and/or antiphospholipid syndrome—a routine 1st trimester ultrasound, with Doppler in the 2nd trimester, and Doppler of the major fetal arteries every month starting at the 3rd trimester [38]. 

Those at risk for NLE through antibody positivity or with a history of an infant with NLE should have recurring fetal echocardiograms, as previously discussed. 

Sjögren’s syndrome and pregnancy carry increased risks for dental caries [46,47]. Therefore, it is doubly important to have timely dental preventive care and follow-up. 

Autoimmune thyroid disease is also associated with autoimmune rheumatic disease [48]. Once pregnant, treatment of hypothyroidism should be titrated to trimester-specific ranges [49]. 

### 4.4. Should I Take a Baby Aspirin during My Pregnancy? 

Patients with SLE, APS, positive aPLs, and systemic vasculitis are at elevated risk for preeclampsia. Anyone with a high risk of preeclampsia should take a low-dose aspirin (81 or 100 mg) daily, according to the US Protective Health Task Force and the American College of Obstetricians and Gynecologists. This can also include those with a prior history of preeclampsia, having a multifetal gestation, or underlying comorbidities such as hypertension, diabetes, renal disease, or obesity [50]. Stemming from this recommendation, patients with SLE, APS, and systemic vasculitis—as well as any other rheumatic patients with elevated preeclampsia risk—can take a baby aspirin to reduce their risk and discontinue around delivery after discussion with their obstetrician and anesthesiologist [1,38,42].

### 4.5. Do I Need a Blood Thinner during Pregnancy? 

If diagnosed with obstetric antiphospholipid syndrome (Ob APS), heparin or low-molecular-weight heparin (LMWH) at a prophylactic dose are recommended to prevent adverse pregnancy outcomes such as pregnancy loss [1,38,41]. Those with Behçet’s disease and a history of thrombosis are also advised to start anticoagulation [42]. LMWH and heparin are not transferred to the fetus via the placenta. The ACR recommends continuation of LMWH until 6–12 weeks postpartum. In thrombotic APS, a full therapeutic dose of heparin or LMWH is recommended in addition to low-dose aspirin. Hydroxychloroquine may also help for those with primary APS. 

### 4.6. If I Want to Become Pregnant, Do I Need to Stop My Medications before I Try to Conceive? If So, When?

There are excellent, clear recommendations published by the American College of Rheumatology (ACR) [1], the British Society for Rheumatology (BSR) [39,41], and the European Alliance of Associations for Rheumatology (EULAR) [40] about the safety of medications prior to, during, and postpartum (if breastfeeding). These include recommendations on when to stop teratogenic medications or those incompatible with pregnancy.

#### 4.6.1. Teratogens

The effect of cyclophosphamide is well-known; this alkylating agent can cause male and female infertility in a cumulative dose-dependent fashion. Its risk for causing premature ovarian failure and oligo- or azoospermia should be discussed prior to use in patients with reproductive potential [1]. Cyclophosphamide should be discontinued at least 3 months before conception. If cyclophosphamide use is planned but has not yet occurred, cryopreservation and/or use of a gonadotropin-releasing hormone (GnRH) agonist should be discussed with a reproductive specialist. Treatment with a GnRH analog, such as leuprolide acetate or triptorelin, can preserve ovarian function and prevent premature ovarian failure [26,51,52]. 

As for other teratogens, mycophenolate should be discontinued 6 weeks prior to trying for pregnancy, and methotrexate 1–3 months prior [1,39,40]. Patients taking methotrexate with or without TNF inhibitors have not been shown to have decreased ovarian reserve [53]. Past methotrexate use has also not been associated with increased time to pregnancy; on the contrary, those with RA who had not experienced infertility were more likely to have used methotrexate [54]. Although there are insufficient human studies of leflunomide in pregnancy, given the animal studies demonstrating teratogenicity, it is recommended to discontinue the drug prior to pregnancy [1,38,39,40,55]. Due to its long half-life, leflunomide levels should be tested prior to attempting pregnancy, and if detectable, cholestyramine washout should be given. 

#### 4.6.2. Other DMARDs

Other conventional synthetic DMARDs, such as hydroxychloroquine, sulfasalazine, azathioprine (up to 2 mg/kg/day), cyclosporine/tacrolimus, and colchicine, can be continued prior to conception and throughout the pregnancy [1,39,40]. TNF inhibitors can be continued past conception through the first and second trimester, but certolizumab—and possibly etanercept, according to EULAR and BSR—can be continued throughout pregnancy. Due to the lack of evidence regarding safety in pregnancy, other biologics (anakinra, canakinumab, tocilizumab, sarilumab, ustekinumab, secukinumab, abatacept, rituximab, and belimumab) and targeted synthetic DMARDs (tofacitinib, baricitinib, ruxolitinib, and apremilast) are recommended to be discontinued at conception, if possible. 

The challenge, however, is that disease activity should be controlled prior to conception in order to reduce the risk of adverse pregnancy outcomes. Therefore, ideally the patient should be transitioned to—and stable on—a pregnancy-safe DMARD prior to pregnancy attempts. 

#### 4.6.3. NSAIDs

Although NSAIDs are safe to take pre-pregnancy, COX-2 selective NSAIDs, such as etoricoxib, may affect ovulation [56]; therefore, the ACR 2020 guidelines have suggested discontinuing these if having difficulty conceiving [1]. BSR and EULAR go so far as to recommend discontinuation of COX-2 selective inhibitors prior to attempting pregnancy [41]. 

#### 4.6.4. Glucocorticoids

Steroids should be tapered to the least effective dose prior to pregnancy (ideally less than 10 mg/day of prednisone or prednisolone) and augmented with DMARDs that are safe in pregnancy [1]. Due to a greater risk of hyperglycemia and hypertension with both steroids and pregnancy, blood glucose levels and blood pressure should be monitored closely [39].

#### 4.6.5. Anticoagulation, Antihypertensives, and Vasodilators

Warfarin, the anticoagulant of choice in patients with antiphospholipid syndrome (APS), should be discontinued prior to conception and patients should be transitioned to low-molecular-weight heparin or fondaparinux [40]. Just as with APS outside of pregnancy, direct oral anticoagulants (DOACs) are not recommended. However, if needed for another indication, DOACs should not be used immediately preceding or during pregnancy. The only DOAC compatible with lactation, according to the BSR guidelines, is rivaroxaban. 

Due to the risk of teratogenicity, ACE inhibitors and ARBs should be discontinued at the time of pregnancy [41]. However, an exception can be made for active scleroderma renal crisis, as the risk of fetal and maternal mortality is greater with untreated disease [1]. The calcium channel blockers often used in scleroderma and Raynaud’s, such as nifedipine and amlodipine, are safe to use [41].

There are not sufficient data on vasodilators such as sildenafil, bosentan, and prostacyclines; however, pulmonary arterial hypertension is often a contraindication for pregnancy due to high mortality risk [1,41].

### 4.7. If I Am a Male with Rheumatic Disease Planning Pregnancy with a Birthing Partner, Do I Need to Stop My Medications? If So, When? 

For male patients with rheumatic disease, the ACR and BSR guidelines also include helpful charts on what medications should be discontinued or continued [1,39]. As with females, cyclophosphamide should be discontinued 12 weeks prior to attempts at pregnancy [1]. Given that sulfasalazine has been shown to affect sperm quality and cause reversible infertility in male patients, it has been advised to discontinue sulfasalazine for three months and test semen if unable to become pregnant for a year [57]. TNF inhibitors (TNFi) appear to be safe to continue for men planning pregnancy: a 2010 study of patients with SpA found that active disease was correlated with worse sperm quality compared to healthy controls, but long-term use of TNFi in those with inactive disease was not [58]. If male fertility is at risk due to chemotherapy agents, such as cyclophosphamide, preservation of fertility should be discussed and planned prior to initiation [1]. Stahl et al. have published an algorithm to assist with cryopreservation [59]. 

## 5. Pregnancy and Neonatal Outcomes

### 5.1. Is There a Greater Risk for Miscarriage or Perinatal Death (Which Includes Stillbirth and Neonatal Death)? 

There may be a greater risk of these for patients with autoimmune connective tissue diseases (CTDs). 

While prior studies did not indicate increased spontaneous abortions in RA, more recent data from a birth registry in Norway showed that a very small increase in the miscarriages was found in patients with RA compared to controls with a relative risk (RR) of 1.2 prior to week 12 and an RR of 1.4 in weeks 12–22 of gestation [60]. A systematic review and meta-analysis published in 2023 also showed a greater risk of stillbirth with an odds ratio (OR) of 1.99 compared to controls [61]. 

A recent study from a South Korean database found an increased risk of death within a year of birth for offspring of women with SLE (2 cases out of 1322 offspring, or 15.1 per 10,000 persons) over age-matched controls (10 deaths in 20,418 offspring, or 4.9/10,000 persons) [62]. The study did not identify a cause; however, in the two deaths, the authors noted that SLE had been diagnosed in each mother 6–8 months prior to pregnancy, and neither had taken hydroxychloroquine prior to, or during, their pregnancy. No deaths were reported in the offspring of patients with seropositive RA or ankylosing spondylitis in this study. Active SLE and lupus nephritis (either a history of or currently active) are both associated with increased pregnancy loss [38].

In a systematic review and meta-analysis by Upala et al., patients with primary Sjögren’s syndrome were found to have increased rates of total fetal loss [63]. Of the drivers of this association, one study included induced abortions, and another study included a patient who had four fetal losses, the exclusion of whom resulted in a statistically insignificant difference. None of the seven studies evaluated were excluded from this meta-analysis for poor quality. However, an increased risk of miscarriage for patients with Sjögren’s syndrome was also found in an umbrella review published as a meeting abstract in *The Lancet* [64].

A retrospective cohort study of patients with rare CTDs—systemic and cutaneous vasculitis, Sjögren’s, systemic sclerosis, Behçet’s, polymyositis/dermatomyositis, and other systemic CTD—found increased incidence of perinatal death (defined as stillbirth or neonatal death) in 15 of the 409 births (3.7% compared to 0.8% in the general population) [65]. In this cohort, severe neonatal morbidity was associated with systemic vasculitis (OR 1.20–2.55) and systemic sclerosis (OR 1.27–2.71), even after adjusting for age. In patients with systemic sclerosis and pulmonary arterial hypertension, the risk of neonatal mortality is high at 11–13% and accompanied by an even higher maternal mortality rate [66].

Patients with systemic vasculitis can experience various pregnancy complications related to their disease such as hypertension, preeclampsia, preterm delivery, spontaneous abortion, and low birthweight [42]. In their comprehensive review published in 2022, Sims and Clowse include a concise, clear table conveying the potential complications associated with each type of systemic vasculitis and the risk factors correlated with these adverse outcomes. Patients with Behçet’s, IgA vasculitis, and small-vessel vasculitides, such as granulomatosis with polyangiitis (GPA), microscopic polyangiitis (MPA), and eosinophilic granulomatous polyangiitis (eGPA), are at greater risk for spontaneous abortion. The authors also included a helpful table with the findings that distinguish active vasculitis from mimickers such as preeclampsia, chronic and gestational hypertension, and HELLP (hemolysis, elevated liver enzymes, and low platelets) syndrome. 

### 5.2. What Is the Likelihood of Other Pregnancy Complications? 

There appear to be increased risks of other pregnancy complications in patients with rheumatic disease. It is unclear why, and many of the studies do not stratify based on disease activity.

For patients with RA, Sim et al. found higher rates of caesarean section (C-section), preterm birth, preeclampsia, and small for gestational age (SGA) in their 2023 systematic review [61]. No difference was found between the groups with respect to congenital abnormalities. Patients with juvenile idiopathic arthritis (JIA) have also been shown to be at increased risk for C-section, but only emergency and not elective C-sections, at a rate of 20.4% compared to that of the controls at 15.6% [67]. A recent study of JIA patients appeared to have similar rates of spontaneous abortion and major congenital anomalies with the general population, although premature birth and C-section rates were higher [68]. A cohort of 238 pregnancies among those with connective tissue diseases and inflammatory arthritides from a registry at a German hospital had rates of pregnancy losses (5%) similar to those of the general population; however, the published report had no controls, patients were 95% Caucasian, and the data were published as a letter to the editor [69].

In the EULAR-published reproductive recommendations specifically for patients with SLE and APS, a checklist (table) shows risk factors and their associated complications [38]. SLE that is active immediately before or at conception is correlated with increased risks for hypertensive disorders of pregnancy such as preeclampsia, intrauterine growth restriction (IUGR), and preterm delivery. Lupus nephritis (whether historical or active) is associated with preterm delivery. APS and high-risk aPLs are associated with preeclampsia, IUGR, and preterm birth. 

The Australian retrospective cohort study of 409 births in patients with rarer CTDs—systemic and cutaneous vasculitis, Sjögren’s, systemic sclerosis, Behçet’s, polymyositis/dermatomyositis, and other systemic CTD—found that some of these diseases were associated with adverse pregnancy outcomes [65]. Those in the study with systemic vasculitis had an increased incidence of hypertension, C-section, and severe maternal morbidity, even when adjusted for maternal age, country, socioeconomic status, pre-existing hypertension, pre-existing diabetes, smoking, and parity. Their offspring were at greater risk for preterm birth (<37 weeks gestation), and severe neonatal morbidity. Cutaneous limited vasculitis did not have significant associations with these complications. With Sjögren’s patients in this cohort, there were higher rates of labor induction, C-section, severe maternal morbidity, preterm birth, and neonatal intensive care unit (NICU) admission. Patients with polymyositis/dermatomyositis also had increased rates of pregnancy hypertension, antepartum hemorrhage, C-section, and preterm birth. Behçet’s disease was also associated with increased antepartum hemorrhage and labor induction. Those with systemic sclerosis were particularly at risk, with higher rates of C-section, postpartum hemorrhage, severe maternal morbidity, preterm birth, NICU admission, and severe neonatal morbidity. Those with any autoimmune disease, Sjögren’s, and Behçet’s had elevated rates of postpartum venous thromboembolism (VTE) (1–3.1%) compared to that of the general obstetric population (0.1%). Notably, no significant differences were found in the birth weights of these offspring. Although a mildly increased rate of major congenital anomalies was found among the 409 births over controls, this did not reach significance. One-third of these anomalies were cardiac disorders, including congenital heart block. In a systematic review and meta-analysis of patients with primary Sjögren’s syndrome, there was no association between Sjögren’s syndrome and premature birth [63].

The 2021 study using a South Korean claims database showed correlations between SLE, seropositive RA, and ankylosing spondylitis (AS) and C-section [62]. SLE was also associated with an increased incidence of threatened abortion, preterm birth, preeclampsia/eclampsia, and IUGR. In this cohort, seropositive RA patients had significant correlations with threatened abortion and IUGR, but not with preterm birth or preeclampsia/eclampsia. AS had no associations with any of these pregnancy complications. 

### 5.3. Is It Safe to Continue Biologics during Pregnancy? 

The majority of studies on biologics and pregnancy outcomes are unable to distinguish whether adverse outcomes are due to biologic exposure or underlying disease activity. The available data are primarily on TNF inhibitors.

A retrospective cohort study published in 2023 using a national claims database in South Korea found an association between biologic use during pregnancy and IUGR, preterm birth, preeclampsia/eclampsia, and C-section [70]. However, this did not account for underlying disease activity, which is independently associated with adverse pregnancy outcomes and may warrant biologic use during pregnancy. The study also found a link between biologic use and decreased risks of fetal loss and gestational diabetes. No difference in live birth rate was found. 

Other studies have suggested that it may be safe to continue TNFi throughout pregnancy [71,72]; however, the ACR, EULAR, and BSR guidelines suggested stopping most TNF inhibitors at some point during pregnancy [1,39,40]. They all agree that certolizumab, a TNFi, is safe to continue without interruption because of negligible placental transfer [73]. Etanercept, a TNFi, may have less placental transfer than other TNF inhibitors, and the EULAR guidelines recommend continuing it through the end of pregnancy [40,71]. Eculizumab, a C5a inhibitor used for eGPA—as well as myasthenia gravis, atypical hemolytic uremic syndrome, and paroxysmal nocturnal hemoglobinuria—may also have little placental transfer; however, more evidence is needed to confirm its safety in pregnancy [71]. Intravenous immunoglobulin (IVIG) is thought to be safe during pregnancy, according to the BSR guidelines, while EULAR recommends reserving its use for refractory disease [39,40]. While rare, IVIG does carry a thromboembolic risk but is used for certain conditions during pregnancy such as severe or refractory immune thrombocytopenia (ITP) or catastrophic APS [74].

There is limited and/or conflicting evidence regarding the safety of other biologics such as abatacept, IL-1 inhibitors (e.g., anakinra, canakinumab, rilonacept), IL-6 inhibitors (e.g., tocilizumab, sarilumab), IL-17 inhibitors (e.g., secukinumab, ixekizumab), IL-12/-23 inhibitors (e.g., ustekinumab), and B cell inhibitors (e.g., belimumab, rituximab) [71]. Therefore, the ACR, EULAR, and BSR have recommended discontinuation prior to conception [1,39,40].

### 5.4. Is It Safe to Continue Other DMARDs or Anti-Rheumatic Drugs during Pregnancy?

The EULAR publication contains a table summarizing the data from 2008–2015 on adverse pregnancy outcomes such as miscarriages and congenital malformations [40]. 

Teratogens, such as cyclophosphamide, mycophenolate, methotrexate, and possibly leflunomide, should have been discontinued well prior to pregnancy, as should other medications such as warfarin and ACE inhibitors [1,39,40,41]. If a patient becomes pregnant while on methotrexate, it should be discontinued immediately and 5 mg daily of folic acid should be initiated. Although human studies of leflunomide have not found a clear pattern of congenital malformations, animal studies have shown teratogenicity; therefore, it has been recommended to discontinue leflunomide by the ACR, EULAR, and BSR [1,39,40,55]. If accidentally pregnant while on leflunomide, stop leflunomide and administer the cholestyramine washout. Exceptions include cyclophosphamide and ACE inhibitors for severe, life-threatening rheumatic disease and active scleroderma renal crisis, respectively. 

Hydroxychloroquine (HCQ), sulfasalazine, azathioprine (up to 2 mg/kg/day), cyclosporine/tacrolimus, and colchicine can be continued throughout the pregnancy [1,39,40]. Substantial evidence supports HCQ use for patients with SLE and those with SSA and/or SSB positivity to prevent neonatal lupus [1,38,39,75]. HCQ has been shown to prevent the feared complication of neonatal lupus—congenital heart block [76]—without a significant risk of birth defects or adverse pregnancy outcomes [77]. In a 2020 study, 5 out of 45 infants whose mothers were treated with 400 mg daily of hydroxychloroquine during pregnancy to prevent congenital heart block recurrence had prolonged QTc, although the clinical significance of these findings is unknown and there was no control group [78]. Among those with major congenital malformations in a small 2021 analysis of two multicenter case control studies, there appeared to be no clear pattern of birth defects, as is often the case with teratogens [79]. A more recent study of 453 infants with possible first-trimester exposure to HCQ showed no elevated risk for major congenital malformations, the caveat being that exposure was counted if a prescription was filled for HCQ in the 3 months prior to pregnancy (given the long half-life of HCQ) and the first trimester [80]. Although HCQ is typically dosed according to weight, a recent study suggested that dose adjustments may not need to be made during pregnancy [81].

There are insufficient data on pregnancy outcomes with the newer small-molecule DMARDs, such as JAK inhibitors (e.g., tofacitinib, ruxolitinib, baricitinib) and PDE-4 inhibitors (e.g., apremilast), which is why the ACR, EULAR, and BSR recommendations currently advise discontinuation prior to conception [1,39,40]. The BSR recommends discontinuing JAK inhibitors at least 2 weeks before conception [39]. The very limited evidence available has found similar rates of adverse pregnancy outcomes to the general population for tofacitinib [82,83]. 

As previously mentioned, COX-2 selective inhibitors are often recommended to be discontinued prior to conception [1,40,41]. All NSAIDs should be stopped by the third trimester to prevent premature closure of the ductus arteriosus [1]. 

### 5.5. Are Vaccinations for My Child Going to Be Less Effective If I Took a Biologic or Small-Molecule DMARD While Pregnant? 

Reports of insufficient response to vaccines after in utero exposure to biologics have been published, with most of the data on exposure to TNF inhibitors [71]. Some children required boosters to produce sufficient serologic response, such as the Hemophilus influenza B (HiB) and diphtheria vaccines; however, a booster is sometimes needed in unexposed children, too. Developmental outcomes appear to be comparable to healthy controls. Limited data exist on children exposed to other biologics and small-molecule DMARDs. If biologics (except certolizumab) are used in the third trimester, the BSR has recommended delaying live-attenuated vaccines such as Bacille Calmette–Guerin (BCG) or rotavirus until at least 6 months of age (39). 

### 5.6. What Long-Term Developmental Risks for My Child Have Been Studied? 

A scoping review of studies on patients with inflammatory arthritis due to RA, JIA, seronegative spondyloarthropathy, or IBD-related arthritis was conducted by Chock et al. in 2023 [84]. It did not find a greater risk for neurodevelopmental outcomes, such as attention deficit hyperactive disorder (ADHD) or autism spectrum disorder (ASD), in those patients who took NSAIDs, TNFi, methotrexate, or glucocorticoids. In a study of the Taiwanese offspring of mothers with SLE or RA, there were also no increased rates of ASD among their offspring compared to controls matched for factors such as age, Apgar scores, and gestational age [85]. 

### 5.7. What Is the Likelihood My Child Will Get My Disease?

This is a difficult question to answer, in part because of how genetic influences on disease pathogenesis is studied. For instance, RA is thought to be 60% heritable [86]. This does not imply that an individual’s chances of inheriting the disease from a parent who has it is 60%, but rather that 60% of the disease is thought to be due to genetic factors. The recurrence risk—or how likely it is that a disease will occur in another family member—is thought to be much lower [87]. Additionally, many of these diseases are not associated with a single gene but are polygenic. The largest proportion of this genetic component for RA is due to the HLA-DRB1 alleles. The greater the number of disease-associated alleles, the greater one’s chances are of developing RA. Certain factors, such as obesity and vitamin D deficiency, mildly increase one’s risk, but perhaps the greatest association with RA development and severity is smoking [88]. Smoking has been shown to increase the risk of developing RA 20 times over that of nonsmokers, even with the same genetic factors. 

SLE may be up to 66% heritable, and onset may be triggered by factors such as ultraviolent light exposure and hormones [89]. Although HLA-B27 is the most well-known association with ankylosing spondylitis, other genes also play a role [90]. AS has been found to have a recurrence risk of about 8% in children [91]. The genetic contributions to other diseases, such as Sjögren’s syndrome, systemic sclerosis, and systemic vasculitides such as ANCA-associated vasculitis, have been harder to elucidate but continue to be studied in genome-wide association studies [92,93,94]. In systemic sclerosis, exposures such as asbestos, silica, and air pollution seem to increase one’s risk of developing the disease; however, it is unknown exactly how [95].

Currently, genetic testing for these diseases is not commonly performed, except in research studies, likely in part due to the polygenic nature and significant non-genetic component in the development of these diseases. It remains unclear how much influence arises from other aspects such as lifestyle factors (such as sleep, stress levels, and diet), prior infections, and environmental factors (such as air pollution) [95]. While autoantibody positivity can predate the development of clinical disease, those with autoantibodies do not always develop disease. Therefore, testing is not recommended in the asymptomatic child. 

## 6. Maternal Outcomes

### 6.1. What Is the Likelihood My Disease Will Flare While I’m Pregnant? 

Greater risk of SLE flare during pregnancy has been found in those with active disease in the 6–12 months before or at conception, serologically active SLE, and lupus nephritis that is either historical or active [38]. The increased risk of an SLE flare if one has ever had lupus nephritis was supported by a 2021 retrospective study [37]. Complement levels have been shown to rise during the first two trimesters but were noted to be lower among those who flared [96]. In this publication, 32% of SLE patients from four studies flared during pregnancy. Stopping hydroxychloroquine increases the risk of flares [38].

RA is classically associated with decreased disease activity during pregnancy, possibly as a side effect of immunotolerance of the fetus [97]. However, flares can still happen and occurred in about a third of patients in a recent German study [98]. Having active disease in the first trimester has been shown to not bode well for disease activity in the third trimester [99]. In this cohort, well-controlled disease in the first trimester, seronegativity, and a lack of prednisone use in the first trimester were positively correlated with remission in the third trimester. 

In a multicenter study of patients with Sjögren’s syndrome, 13% flared during pregnancy [100]. Patients with systemic sclerosis can sometimes experience exacerbations of their gastrointestinal and pulmonary symptoms, and spirometry and oxygen saturation can be obtained to assist in management [66]. Raynaud’s symptoms can improve during pregnancy, but skin-thickening can worsen postpartum. In patients with vasculitis, increased flares tend to be associated with recently diagnosed or active disease [101]. About a quarter of pregnant patients with axSpA in a small recent study were found to flare [98]. Once again, active disease at the beginning of pregnancy portended later flares.

Stopping DMARDs can also be associated with a flare. Although biologic exposure during pregnancy has been correlated with increased flare rates postpartum, discontinuing a biologic before pregnancy is linked to active disease (at twice the rate), gestational diabetes, steroid use, and flare—all risk factors for adverse pregnancy outcomes [102]. In this retrospective cohort study of patients with AS, RA, psoriatic arthritis (PsA), and IBD, those who discontinued their biologic within 3 months of conception had active disease at twice the rate of those who continued the biologic throughout pregnancy.

### 6.2. If I Do Experience a Flare, What Medications Can I Take?

Non-selective NSAIDs can be used during pregnancy but should be avoided during the third trimester to ensure that the ductus arteriosus remains open for fetal oxygenation [1,40,41].

Although glucocorticoids can be taken during pregnancy, non-fluorinated oral steroids (prednisone, prednisolone) are recommended at the minimum effective dose [1,39,40]. Fluorinated steroids (betamethasone, dexamethasone) are typically avoided given their passage to the fetus in their fully active form, whereas non-fluorinated steroids undergo metabolization in the placenta, reducing the amount of active drug available to the fetus. Methylprednisolone, a potent IV non-fluorinated steroid, as well as IVIG and plasmapheresis, can be used in severe intractable disease [38]. 

### 6.3. Do I Need Stress-Dose Steroids? 

As with any other major surgery, stress-dose steroids can be considered for C-sections after discussion with the obstetrician and anesthesiologist but are generally not required in vaginal deliveries [1].

### 6.4. Am I at Risk for Any Complications? 

In the Australian study of 409 births among mothers with rare CTDs, there were slightly longer hospital admissions (6.5 vs. 4.9 days) and more hospital admissions before and after the birth [65]. Postpartum hemorrhage occurred with greater frequency in patients with systemic sclerosis, and severe maternal morbidity was increased among those with Sjögren’s, systemic vasculitis, and systemic sclerosis. Postpartum VTE occurred in 3% of Sjögren’s patients and 3% of Behçet’s patients. 

Patients with APS or high-risk aPLs also have an increased risk of thrombotic events [38]. Both primary APS and SLE are associated with an increased risk of complications associated with hypertensive disorders of pregnancy such as preeclampsia and HELLP syndrome. 

Patients with vasculitis can also experience various adverse outcomes related to their disease such as hemorrhage, renal injury, and hypertensive complications [42]. Steps should be taken to control hypertension and disease activity to prevent these complications. 

Due to significant maternal mortality risk, estimated to be 17–33%, pregnancy is advised against for those with systemic sclerosis complicated by pulmonary arterial hypertension [66]. Systemic sclerosis patients with interstitial lung disease and a forced vital capacity (FVC) of under 50% predicted are also advised by some to avoid pregnancy. 

## 7. Postpartum

### 7.1. Are My Meds Still Going to Work If I Stopped Them during Pregnancy? 

Patients who interrupt treatment of autoimmune diseases with biologics can sometimes experience loss of efficacy [103,104]. In a study of Dutch patients with RA, SpA, PsA, JIA, and other inflammatory arthritides, over 80% of patients who stopped their biologic or methotrexate during pregnancy were still on the same DMARD a year and 3 years later [105]. The most common reason patients discontinued their DMARD was another pregnancy. Although this does not confirm that the original DMARD remained effective for the patients, it suggests that this may be the case. 

### 7.2. Which Medications Are Safe in Breastfeeding?

The EULAR publication contains charts summarizing data from 1970–2015 on lactation (i.e., how much of the drug is found in breast milk and in infants and what side effects—or lack thereof—have been noted in breastfed children) [39]. 

Hydroxychloroquine, sulfasalazine, azathioprine (up to 2 mg/kg/day), cyclosporine/tacrolimus, colchicine, IVIG, and nonselective COX-inhibitors can be continued while breastfeeding [1,39,40]. The ACR specifically recommends ibuprofen as the NSAID of choice. 

Certolizumab, which is also safe and recommended in pregnancy, is also safe in breastfeeding given the lack of transfer into breast milk [106]. Other TNF inhibitors are also likely to be safe [40].

There is insufficient information regarding many other DMARDs in lactation. The ACR, EULAR, and BSR all agree that methotrexate, mycophenolate mofetil, leflunomide, and cyclophosphamide should be withheld during breastfeeding [1,39,40]. However, the 2020 ACR guidelines conditionally recommend the use of anakinra, belimumab, abatacept, tocilizumab, secukinumab, and ustekinumab during lactation due to their large molecule size (and the 2023 BSR guidelines agree), whereas the EULAR guidelines, published earlier in 2016, recommend avoidance due to the lack of data on transfer to breast milk. Targeted synthetic DMARDs, like JAK inhibitors (tofacitinib, baricitinib) and PDE-4 inhibitors (apremilast), have not been recommended by all three organizations, given the lack of information. The ACR and BSR consider rituximab safe to resume postpartum, even during lactation; however, EULAR recommends against its use during lactation due to the lack of data.

If switched from warfarin to heparin or LMWH during pregnancy, warfarin can be resumed, as the BSR considers it compatible with breastfeeding [41]. 

The National Library of Medicine maintains a database called LactMed^®^, which publishes known data about the safety of drugs in lactation [107]. Previously, a LactMed^®^ app was available, but this was discontinued in 2019 and has been replaced by the LactRx app by MotherToBaby, which is free to use and accesses the same LactMed^®^ database [108].

## 8. Conclusions

Planning a pregnancy can be fraught, but even more so with an autoimmune rheumatic disease and its complications. Synthesizing the available data can be difficult for busy clinicians with many competing priorities, and optimal management based on published guidelines often does not occur. For instance, low-dose aspirin was used in only 25% of a cohort of SLE patients (some of whom had other risk factors such as lupus nephritis and antiphospholipid antibodies), despite the recommendations by multiple organizations for its administration in cases of high preeclampsia risk [109]. There are still high rates of unplanned pregnancies among high-risk patients, and contraceptive counseling can be lacking in the context of additional obstacles such as a language barrier [110]. Frustratingly, higher rates of adverse pregnancy outcomes still exist among people of color and marginalized populations [111]. Reduced access to specialized care is another significant challenge, particularly in nonurban areas [112]. At times, even reticence from patients in discussing reproductive matters can get in the way. Formal institutional protocols are scarce in the care of pregnant patients with rheumatic disease, and little evidence exists to guide providers on how to proceed with rarer diseases such as IgG4-related disease, idiopathic inflammatory myopathies, relapsing polychondritis, and Behçet’s disease [113]. Therefore, the quality of reproductive care can be extremely mixed and is apt to vary from provider to provider. 

Access to quality reproductive care for rheumatic patients makes a difference. In addition to the mountain of evidence linking poorly controlled disease with adverse pregnancy outcomes, there are also data to suggest that quality pre-pregnancy planning is associated with fewer flares, fewer changes in medication while pregnant, and lower disease activity in the first trimester [114].

It is heartening to see the focused efforts underway by clinicians and researchers all over the globe. In addition to the official ACR, EULAR, BSR, ACOG, and other organizational guidelines for the care of individual patients, there are models for institutions, such as the formation of multidisciplinary care clinics [115], and suggestions for formal pathways of care [112,113]. A focus group of rheumatologists and advanced care practitioners has advised priming patients to discuss reproductive health, having efficient referral systems to obstetric and maternal-fetal-medicine providers, and allowing for sufficient access to quality training and education on this topic [116]. To this end, the creation of this publication is to assist any provider seeking to better their care. 

For those supporting patients with rheumatic disease on their reproductive journey, having early and frequent discussions about reproductive health, collaborating with our colleagues in other specialties, altering medications to be compatible, and treating with the goal of remission prior to pregnancy are all tools we have in our toolkit. While currently used by providers to varying degrees [117], these tools empower our patients to take steps toward their reproductive goals with the information they so desperately seek.
